# CD5 levels define functionally heterogeneous populations of naïve human CD4^+^ T cells

**DOI:** 10.1002/eji.202048788

**Published:** 2021-03-19

**Authors:** Aditi Sood, Marie‐Ève Lebel, Mengqi Dong, Marilaine Fournier, Suzanne J. Vobecky, Élie Haddad, Jean‐Sébastien Delisle, Judith N. Mandl, Nienke Vrisekoop, Heather J. Melichar

**Affiliations:** ^1^ Immunology‐Oncology Unit Maisonneuve‐Rosemont Hospital Research Center Montreal Quebec Canada; ^2^ Département de Microbiologie, Infectiologie et Immunologie Université de Montréal Montreal Quebec Canada; ^3^ Service de Chirurgie Cardiaque Centre Hospitalier Universitaire Sainte‐Justine Montreal Quebec Canada; ^4^ Département de Pédiatrie Université de Montréal, Centre Hospitalier Universitaire Sainte‐Justine Montreal Quebec Canada; ^5^ Département de Médecine Université de Montréal Montreal Quebec Canada; ^6^ Department of Physiology McGill University Montreal Quebec Canada; ^7^ Department of Respiratory Medicine Center for Translational Immunology University Medical Center Utrecht Utrecht The Netherlands

**Keywords:** Cytokines, CD4^+^ T cells, CD5, Human T cells, Thymus

## Abstract

Studies in murine models show that subthreshold TCR interactions with self‐peptide are required for thymic development and peripheral survival of naïve T cells. Recently, differences in the strength of tonic TCR interactions with self‐peptide, as read‐out by cell surface levels of CD5, were associated with distinct effector potentials among sorted populations of T cells in mice. However, whether CD5 can also be used to parse functional heterogeneity among human T cells is less clear. Our study demonstrates that CD5 levels correlate with TCR signal strength in human naïve CD4^+^ T cells. Further, we describe a relationship between CD5 levels on naïve human CD4^+^ T cells and binding affinity to foreign peptide, in addition to a predominance of CD5^hi^ T cells in the memory compartment. Differences in gene expression and biases in cytokine production potential between CD5^lo^ and CD5^hi^ naïve human CD4^+^ T cells are consistent with observations in mice. Together, these data validate the use of CD5 surface levels as a marker of heterogeneity among human naïve CD4^+^ T cells with important implications for the identification of functionally biased T‐ cell populations that can be exploited to improve the efficacy of adoptive cell therapies.

## Graphical abstract

CD5^lo^ and CD5^hi^ naïve human CD4^+^ T cells differ in their gene expression profiles, affinity for foreign antigen, cytokine production after activation, and accumulation in the memory compartment. These findings have important implications in the identification of functionally biased T‐cell populations that can be exploited to improve cell therapies.

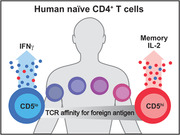

## Introduction

The TCR repertoire is established during T‐cell development in the thymus. A broad range of low to moderate TCR affinities for self‐peptide presented by major histocompatibility complex molecules (pMHC) support positive selection, promoting the survival and differentiation of thymocytes with functional antigen receptors [1]. Positive selection leads to significant variation in self‐reactivity among individual cells within the mature naïve T‐cell population [2]. Subthreshold interactions with self‐pMHC are also necessary for the maintenance and survival of naïve T cells in peripheral lymphoid organs [3, 4, 5, 6], and it is becoming increasingly clear that differences in the strength of these tonic TCR signals among individual T cells, at least in mice, influence their response during subsequent antigen challenge [7, 8, 9, 10, 11, 12, 13].

It has been generally thought that naïve CD4^+^ and CD8^+^ T cells are homogeneous populations with each cell equivalent in their potential to contribute a set of specialized skills to the adaptive immune response. However, recent studies suggest that the strength of tonic TCR interactions with self‐pMHC influences the contribution of individual murine T cells to an immune response upon antigen challenge [7, 8, 9, 10, 11, 12, 13]; this is particularly apparent in the CD4^+^ T‐cell lineage. For example, it has been shown that naïve murine CD4^+^ T cells with higher self‐reactivity also bind with higher affinity to foreign‐pMHC [9, 14]. The higher affinity for their cognate antigen also leads to an increased contribution of these cells during acute antigen challenge in vivo and their subsequent predominance in the memory T‐cell pool [9]. Moreover, the strength of tonic TCR signals is associated with distinct effector fates; in the CD4^+^ T‐cell compartment, murine T cells with higher self‐reactivity have a greater propensity to differentiate into immunosuppressive T_reg_ cells [8, 10], whereas a greater proportion of interferon‐gamma (IFN‐γ) producing CD4^+^ T cells originate from the naïve T‐cell population that experiences lower tonic TCR signals [12]. Further, gene expression differences that are indicative of, at least in part, functional heterogeneity in naïve murine CD4^+^ T cells have also been reported based on the strength of TCR self‐reactivity [15, 16]. While these studies highlight the functional heterogeneity that exists among naïve CD4^+^ T cells in mice, whether naïve human CD4^+^ T cells also possess cell‐intrinsic functional biases remains unclear.

Several markers have been used as a read‐out for the strength of TCR signaling among murine naïve T cells including CD5, Ly6C, and Nur77. CD5 is a negative regulator of antigen receptor signaling expressed by all T cells, and its cell surface levels are directly proportional to self‐ligand reactivity and TCR signal strength [17, 18]. In contrast, Ly6C expression is inversely associated with the strength of TCR interactions with self‐pMHC by naïve murine CD4^+^ T cells [10, 15]. Both CD5 and Ly6C are cell surface markers that have been used to isolate functionally distinct naïve CD4^+^ T‐cell subsets [8, 9, 10, 11, 12, 13]. The relative levels of Nur77, as assessed using Nur77‐GFP reporter transgenic mice, have also been used to identify cells with varying levels of basal TCR signals for functional analyses [19, 20, 21]. In fact, in mice, it was recently shown that a combination of Nur77‐GFP and Ly6C allow for the isolation of naïve CD4^+^ T cells with a greater dynamic range of basal TCR signaling than any one marker individually [22]. Yet, while Nur77 as a read‐out of antigen receptor signaling has been recently confirmed using human samples [23], it is not feasible to use this marker to isolate human T cells for functional tests given its intranuclear localization. In addition, Ly6C cannot be used to delineate human T‐cell heterogeneity as it does not have a human ortholog. Thus, CD5 may be the only viable marker among these for predicting functional heterogeneity among human naïve T cells.

Here, we investigate whether CD5 is a marker of TCR signal strength in human T cells and whether measures of CD5 surface levels can be used to predict the contribution of individual naïve CD4^+^ T cells to an effector response. Indeed, we show that, similar to their murine counterparts, CD5 expression on human naïve CD4^+^ T cells can be used to identify subpopulations with biased functional potentials in terms of antigen reactivity and effector skewing. Predicting the functional capabilities of individual human T cells in immune responses may be useful in directing immunomodulatory therapies to select or preferentially target the most reactive cells.

## Results

### Modulation of CD5 expression on murine and human T cells

**Figure 1:**
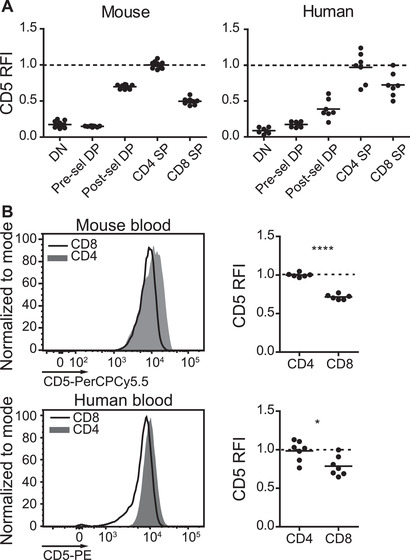
**Regulation of CD5 expression during mouse and human T‐cell development**. (A) Relative fluorescence intensity (RFI) of CD5 on live CD4^−^CD8^−^ double negative (DN), CD4^+^CD8^+^CD69^−^ preselection (pre‐sel) double positive (DP), CD4^+^CD8^+^CD69^+^ postselection (post‐sel) DP, and TCRβ^high^ (mouse) or TCRαβ^high^ (human) Foxp3^−^CD25^−^ CD4^+^ and CD8^+^ single‐positive (SP) thymocytes. Data are normalized to the average mean fluorescence intensity (MFI) of CD5 of CD4^+^ SP cells in each individual experiment. Dots indicate individual mouse (n = 9) or human (n = 7) thymus samples from a minimum of three independent experiments. (B) Representative histogram and RFI of CD5 on naïve CD4^+^ (CD44^−^CD62L^+^CD25^−^Foxp3^−^) and CD8^+^ (CD44^−^CD62L^+^) T cells from mouse blood and human PBMCs (CD45RA^+^CD62L^+^). Dots indicate individual mouse (n = 6) or human (n = 7) blood samples from a minimum of two independent experiments. Data are normalized to the average CD5 MFI of CD4^+^ T cells in each experiment. All data were measured by flow cytometry. Horizontal lines on dot plots indicate average. *p < 0.05 and ****p < 0.0001 as determined by a paired Student's *t*‐test (two‐tailed).

A stepwise, TCR signal‐dependent upregulation of CD5 expression is evident during murine T‐cell development [17, 24]. Immature CD4^−^CD8^−^ double‐negative (DN) thymocytes express low levels of CD5 prior to TCR rearrangement and expression. For conventional T cells, CD5 is upregulated after β‐selection at the transition from the DN to the CD4^+^CD8^+^ double‐positive (DP) stage and further increases after positive selection and differentiation into CD4^+^ or CD8^+^ single‐positive (SP) T cells (Fig. 1A and Supporting Information Fig. S1) [17, 24]. CD5 levels are higher on both CD4^+^ SP thymocytes as well as mature peripheral naïve CD4^+^ T cells than on the comparable CD8^+^ T‐cell populations in mice (Fig. 1A and B and Supporting Information Fig. S2A) [17, 24]. We evaluated whether this also holds true for human T‐cell developmental intermediates. Indeed, analysis of the relative fluorescence intensity (RFI) of CD5 on human thymocytes followed a similar trend of differentiation stage‐dependent upregulation of CD5, with higher levels of CD5 on CD4^+^ as compared to CD8^+^ SP thymocytes and mature peripheral naïve T cells (Fig. 1A and B and Supporting Information Fig. S1 and 2A). Of note, the breadth of CD5 expression on the naïve CD4^+^ T‐cell population, as measured by the coefficient of variation (CV), is significantly narrower in human PBMCs than murine samples originating from either the spleen or peripheral blood (Fig. 1B and Supporting Information Fig. S2B). Importantly, this difference seems to be established during T‐cell development in the thymus as CD5 levels on individual CD4^+^ SP thymocytes from murine samples are already broader than their human counterparts (Supporting Information Fig. S2B).

**Figure 2:**
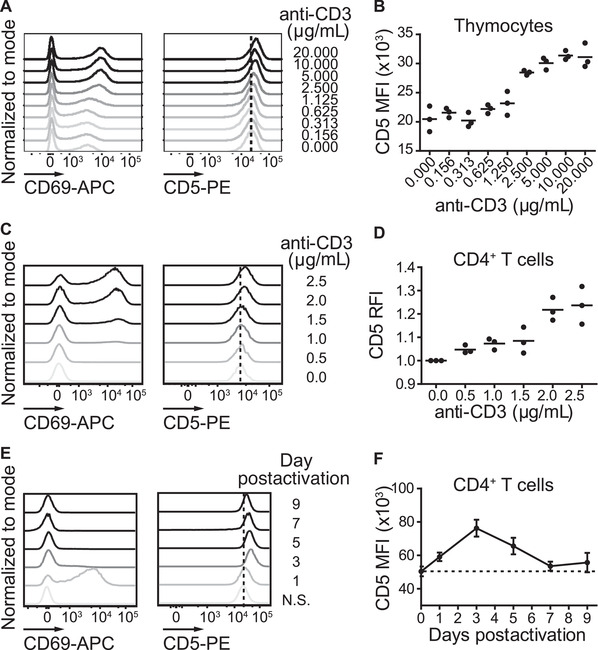
**TCR dose–dependent upregulation of CD5 in human CD4^+^ thymocytes and peripheral blood T cells**. (A) Representative histograms of CD69 (left panel) and CD5 (right panel) levels on human thymocytes activated with the indicated concentrations of anti‐CD3 gated on live CD4^+^ (for CD69) or live CD69^+^CD4^+^ (for CD5) thymocytes 18 h postactivation. (B) MFI of CD5 gated on live CD69^+^CD4^+^ human thymocytes activated with the indicated concentrations of anti‐CD3 18 h postactivation. Dots represent data from technical triplicates from an individual donor. Data are representative of one experiment from three independent experiments. (C) Representative histograms of CD69 (left panel) on live CD45RA^+^CD27^+^ and CD5 (right panel) levels on live CD45RA^+^CD27^+^CD69^+^CD4^+^ T cells from human naïve CD4^+^ T cells stimulated with the indicated concentrations of anti‐CD3 for 24 h. (D) RFI of CD5 gated on live CD45RA^+^CD27^+^CD69^+^CD4^+^ T cells from human naïve CD4^+^ enriched from PBMCs activated with the indicated concentrations of anti‐CD3 24 h postactivation. Data are normalized to the average CD5 MFI of nonactivated naïve CD4^+^ T cells for each experiment. (E) Representative histograms of CD69 (left panel) and CD5 (right panel) levels on live CD4^+^ T cells from naïve CD4^+^ T cells enriched from PBMC and activated with 2.5 μg/mL anti‐CD3. Activated (CD69^+^) T cells were sorted one day postactivation, and cells were rested in the absence of TCR stimulation. (F) MFI of CD5 over time on live CD4^+^ T cells from naïve CD4^+^ T cells enriched from PBMC (Day 0), activated with 2.5 μg/mL anti‐CD3, and sorted (CD69+) (Day 1), prior to resting in the absence of TCR stimulation. (C and D) Dots represent average of individual donors (n = 3) activated in duplicate. Data are from three independent experiments. (E and F) Data are from six individual donors from two independent experiments. All data were measured by flow cytometry. Horizontal lines on dot plots indicate average. Error bars indicate standard error of the mean. N.S., nonstimulated.

Previous studies have shown that CD5 expression on murine T cells is regulated by the strength of TCR signaling, as is illustrated by a dose‐dependent increase in CD5 surface levels when immature thymic T cells are stimulated with increasing concentrations of anti‐CD3 [17]. Therefore, we asked if CD5 expression on human CD4^+^ SP thymocytes also correlates with the strength of TCR stimulation. We cultured murine and human thymocytes with varying amounts of anti‐CD3 and evaluated CD5 induction on activated CD69^+^CD4^+^ SP cells. An anti‐CD3 dose–dependent upregulation of CD69 expression was evident upon T‐cell stimulation (Fig. 2A). Moreover, we observed an upregulation of CD5 for both mouse and human CD69^+^CD4^+^ SP thymocytes postactivation with increasing anti‐CD3 concentrations (Fig. 2A and B and Supporting Information Fig. S3). Notably, within the heterogeneous thymocyte population, multiple developmental intermediates might respond to anti‐CD3 to influence CD5 levels on CD4^+^ SP thymocytes. Therefore, we evaluated whether CD5 expression on mature naïve human CD4^+^ T cells also correlated with the extent of TCR stimulation. Naïve human CD4^+^ T cells were enriched from PBMCs, cultured with varying amounts of anti‐CD3, and CD5 levels on CD69^+^CD4^+^ T cells assessed after activation. Similar to their immature CD4^+^ SP thymocyte counterparts, we observed an anti‐CD3 dose‐dependent upregulation of the proportion of CD69^+^ cells (Fig. 2C and Supporting Information Fig. S4A) as well as a correlation between TCR signal strength and CD5 expression on mature, naïve CD4^+^ T cells from peripheral blood (Fig. 2C and D).

It has been suggested in murine models that CD5 upregulation after activation is transient and returns to basal levels after activation [9]. To determine the extent to which CD5 returns to preactivation levels after TCR stimulation on human CD4^+^ T cells, we activated enriched naïve CD4^+^ T cells from human PBMCs with anti‐CD3, sorted activated (CD69^+^) T cells after 1 day, and then maintained the cells in culture in the absence of further TCR stimulation for several days (Supporting Information Fig. S4B and C). At approximately 1 week after activation, we observed CD5 levels return to similar levels as the enriched naïve CD4^+^ T‐cell population prior to activation (Fig. 2E and F). These data suggest that human CD5 upregulation is transient and TCR dose dependent.

### CD4^+^ T‐cell self‐reactivity directly correlates with strength of binding to foreign antigen

**Figure 3:**
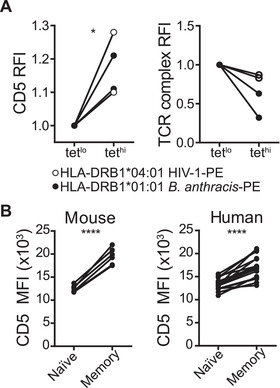
**Human CD4^+^ T‐cell self‐reactivity correlates with strength of binding to foreign antigen and memory recruitment**. Human PBMCs were stained with BV421‐ and PE‐labeled tetramers and cell surface antibodies followed by PE enrichment of tetramer^+^ cells. BV421^+^PE^+^ double‐positive naïve CD45RA^+^CD27^+^CD4^+^ T cells were gated on the top (tet^hi^) and bottom (tet^lo^) 50% of PE‐tetramer staining intensity, and cell surface levels of CD5 and TCR or CD3 expression analyzed by flow cytometry. (A) RFI of CD5 (left panel) or the TCR complex (right panel) on tet^lo^ and tet^hi^ cells is shown for HIV‐1 and *B. anthracis* peptide loaded tetramers for two HLA‐DRB1*04:01 and HLA‐DRB1*01:01 donors, respectively. Data are from four independent experiments with two experiments for each tetramer. Dots represent individual donors (n = 4). RFI was calculated by normalizing to the MFI of CD5 or TCR complex molecules on tet^lo^ cells for each condition. (B) MFI of CD5 on naïve and memory CD4^+^ T cells from mouse blood (left panel) and human PBMCs (right panel). Naïve cells were gated as CD44^−^CD62L^+^CD25^−^Foxp3^−^ for mouse and CD45RA^+^CD27^+^ for human cells. Memory cells were gated as CD44^+^ for mouse and CD45RA^−^ for human cells. Data are representative of a minimum of two independent experiments with a minimum of three individual samples for each experiment. *p < 0.05 and ****p < 0.0001 as determined by paired Student's *t‐* test (two‐tailed).

CD5 expression in murine naïve CD4^+^ T cells correlates with the strength of binding to foreign‐pMHC, as read‐out by tetramer staining intensity [9, 14]. To determine if this is also the case in human naïve CD4^+^ T cells, we examined the relationship between the MFI of CD5 and the binding strength of specific pMHC tetramers (measured by tetramer MFI) [25]. To obtain measurable frequencies of naïve human CD4^+^ T‐cell precursors specific for foreign‐pMHC, we chose an HLA‐DRB1*01:01 restricted peptide from the protective antigen of *Bacillus anthracis* and an HLA‐DRB1*04:01 restricted peptide from P24 gag of HIV‐1; cells specific for these pMHC are present at approximately 6‐10 cells per million total CD4^+^ T cells in healthy donors [26, 27]. In addition, blood donors are unlikely to have encountered these antigens ensuring that all tetramer^+^ T cells are truly naïve. We identified a positive trend between CD5 and tetramer MFI on naïve CD4^+^ T cells for both tetramers tested (Fig. 3A and Supporting Information Fig. S5). Importantly, the higher levels of CD5 staining on tetramer^hi^ naïve CD4^+^ T cells are not due to increased levels of TCR complex components as compared to their tetramer^lo^ counterparts (Fig. 3A and Supporting Information Fig. S5D). Collectively, our data show that higher CD5 levels are associated with greater tetramer staining intensity for both the tetramers tested in a total of four donors, suggesting that, as in mice, CD5 levels on human naïve CD4^+^ T cells are indicative of their affinity for foreign peptide.

In mice, it has been suggested that the daughter cells of naïve CD5^hi^ CD4^+^ T cells with relatively higher affinity for foreign antigen predominate among the memory T‐cell compartment [9]. As such, CD5 expression on murine memory CD4^+^ T cells is greater than on the naïve CD4^+^ T‐cell population (Fig. 3B) [9]. Given the correlation of CD5 levels with tetramer staining intensity on naïve human CD4^+^ T cells, we sought to determine if the human memory T‐cell compartment is also comprised of CD4^+^ T cells with relatively higher levels of CD5. Indeed, the levels of cell surface CD5 on memory human CD4^+^ T cells are significantly higher than their naïve CD4^+^ T‐cell counterparts (Fig. 3B). Given that CD5 levels on murine CD4^+^ T cells with a fixed TCR clonotype remain unchanged by transition from naïve to memory cells, and that CD5 levels return to preactivation levels after TCR stimulation on human CD4^+^ T cells (Fig. 2E and F) [9], our data suggest that high‐affinity human CD4^+^ T cells may be preferentially selected from the naïve T‐cell pool for recruitment into the memory T‐cell compartment and/or preferentially maintained as memory T cells.

### CD5^lo^ and CD5^hi^ naïve human CD4^+^ T cells differ in their gene expression profiles

**Figure 4:**
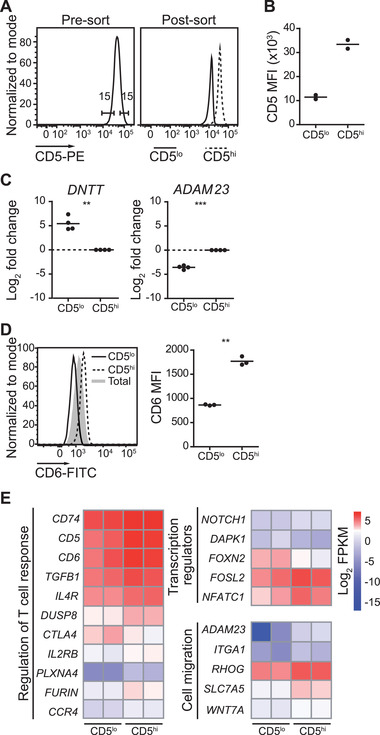
**Differential gene expression among naïve CD5^lo^ and CD5^hi^ CD4^+^ human T cells**. (A) Representative histograms of CD5 levels on pre‐ and postsort naïve CD45RA^+^CD27^+^CD25^−^ CD4^+^ T cells gated on the top and bottom 15% of CD5 expression and analyzed by flow cytometry. (B) MFI of CD5 on sorted cells submitted for RNAseq analysis. Each dot represents an individual donor (n = 2). (C) Analysis of differential gene expression in CD5^lo^ and CD5^hi^ cells by qPCR. cDNA was prepared from naïve CD4^+^ T cells sorted on CD5 expression (top and bottom 20%) and *DNTT* (left panel) and *ADAM23* (right panel) gene expression was analyzed. Average *Ct* values from technical replicates were used for analysis. Target gene expression levels are normalized to internal control gene *HPRT*, and the fold change, calculated as log_2_‐transformed 2^delta‐delta *Ct* values, between the CD5^lo^ and CD5^hi^ subsets is shown. Dots represent individual donors (n = 4). (D) Difference in cell surface CD6 levels on CD5^lo^ and CD5^hi^ cells analyzed using flow cytometry. Representative histogram of CD6 (left panel) and MFI of CD6 (right panel) gated on top and bottom 15% of CD5 expression on naïve CD45RA^+^CD27^+^CD4^+^ T cells. Dots indicate individual donors (n = 3) from two independent experiments. (E) Heat map of select DEG between naïve human CD5^lo^ and CD5^hi^ CD4^+^ T cells enriched within the indicated Gene Ontology pathways at a *p*adj value cut‐off of ≤0.1. Color coding is based on log_2_‐transformed FPKM values. Horizontal lines on dot plots indicate average. **p < 0.01, ***p < 0.001 as determined by paired Student's *t*‐test (two‐tailed).

Microarray analysis of murine CD5^lo^ and CD5^hi^ naïve CD4^+^ T cells has revealed gene expression differences that may contribute to the phenotypic and functional heterogeneity observed between these populations [16]. This is further supported by the observation that naïve Ly6C^−^ and Ly6C^+^ CD4^+^ T cells display distinct gene signatures associated with T_reg_ differentiation biases between these subsets [15]. However, whether variation in gene expression exists with the naïve human CD4^+^ T‐cell population of an individual is unknown. To address this, we performed RNAseq to compare the transcriptional profiles of human CD5^lo^ and CD5^hi^ naïve CD4^+^ T cells from two donors. Cryopreserved human PBMCs were thawed and CD45RA^+^CD27^+^CD25^−^CD4^+^ T cells sorted based on CD5 expression (top and bottom 15%) (Fig. 4A and Supporting Information Fig. S6) such that there was a threefold difference in the MFI of CD5 between CD5^lo^ and CD5^hi^ cells postsort (Fig. 4A and B). Despite significant heterogeneity among donors, our RNAseq analysis revealed that 64 genes were significantly differentially expressed in CD5^lo^ and CD5^hi^ cells (at a Benjamini–Hochberg adjusted *p*‐value [*p*adj] cut‐off of ≤0.05 and foldchange of ≥2; 47 genes were upregulated and 17 downregulated in CD5^hi^ cells as compared to CD5^lo^ cells) (Table 1). Of note, these subtle differences in gene expression between human naïve CD5^lo^ and CD5^hi^ CD4^+^ T cells are consistent with observations in mice for both the CD4^+^ or CD8^+^ T‐cell lineage [7, 15, 16]. Further, in line with these studies, our data showed that more genes are more highly expressed in CD5^hi^ as compared to CD5^lo^ cells.

**Table 1:** Differential gene expression between naïve CD5^lo^ and CD5^hi^ CD4^+^ human T cells

Up in CD5^hi^	Gene symbol	Fold change	*p*adj
1	*ZNF462*	97.64	0.0089
2	*ADAM23*	45.55	0.0441
3	*RP11‐291B21.2*	18.94	<0.0001
4	*ANK1*	14.87	0.0103
5	*CD8A*	11.31	<0.0001
6	*ITGA1*	9.41	0.0301
7	*HPGD*	7.73	0.0062
8	*PLXNA4*	7.21	0.0388
9	*NPAS2*	4.77	0.0191
10	*ERVH48‐1*	4.60	<0.0001
11	*KLF4*	4.42	0.0231
12	*MATK*	4.24	0.0005
13	*C17orf107*	3.78	0.0008
14	*SEPT11*	3.50	0.0001
15	*NKX3‐1*	3.31	0.0251
16	*FAM83F*	3.03	0.0488
17	*CCR4*	3.03	0.0032
18	*CD5*	2.86	<0.0001
19	*SLC7A5*	2.67	<0.0001
20	*NFATC1*	2.66	0.0014
21	*PMEPA1*	2.52	0.0001
22	*BHLHE40*	2.47	0.0405
23	*FURIN*	2.41	0.0032
24	*CD6*	2.38	0.0011
25	*CHKA*	2.37	0.0436
26	*COL18A1*	2.37	0.0021
27	*NOTCH1*	2.34	0.0136
28	*TSHZ1*	2.31	0.0042
29	*CDKN1A*	2.29	0.0003
30	*SEC14L1*	2.26	0.0286
31	*BRD1*	2.24	0.0030
32	*PLAUR*	2.21	0.0341
33	*SLC7A1*	2.20	0.0198
34	*FOSL2*	2.18	0.0018
35	*SKI*	2.17	0.0131
36	*RHOG*	2.14	0.0050
37	*CD74*	2.12	0.0003
38	*NINJ1*	2.12	0.0084
39	*GPR132*	2.07	0.0016
40	*LMNA*	2.03	0.0058
41	*IL4R*	2.02	0.0055
42	*DUSP8*	2.01	0.0316
43	*ORAI2*	2.01	0.0488
44	*PPP1R9B*	2.01	0.0276
45	*RARA*	2.00	0.0074
46	*FBXO34*	1.96	0.0170
47	*EHD4*	1.93	0.0128

Down in CD5^hi^	Gene symbol	Fold change	*p*adj
1	*DNTT*	36.29	<0.0001
2	*ANKRD36BP2*	22.44	<0.0001
3	*DAPK1*	7.36	0.0504
4	*MMRN1*	6.35	0.0348
5	*SLC14A1*	6.33	0.0089
6	*ARRDC3*	3.51	0.0001
7	*MAN2A1*	3.14	<0.0001
8	*TXNIP*	2.95	<0.0001
9	*FOXN2*	2.92	0.0006
10	*ADTRP*	2.70	<0.0001
11	*CTLA4*	2.59	0.0050
12	*ZNF518A*	2.39	0.0048
13	*CASP8AP2*	2.38	0.0415
14	*PREPL*	2.29	0.0077
15	*GVINP1*	2.25	0.0005
16	*CTC‐534A2.2*	2.13	0.0339
17	*SYTL3*	2.08	0.0339

Differentially expressed genes (DEG) up‐ or downregulated in CD5^hi^ CD4^+^ T cells are shown. DEG were defined as those genes that showed statistically significant (*p*adj ≤ 0.05), ≥twofold changes between naïve CD5^lo^ and CD5^hi^ CD4^+^ T cells.

We validated the RNAseq dataset by selectively confirming differential gene expression in naïve CD5^lo^ and CD5^hi^ (top and bottom 20%) CD4^+^ T cells from four independent donors by quantitative RT‐PCR (qPCR). In particular, we assessed the transcript levels of two of the most differentially expressed genes (DEG) among the CD5^lo^ and CD5^hi^ CD4^+^ T cells in our RNA seq analysis: *DNTT* (downregulated in CD5^hi^; log_2_ fold change 5.18) and *ADAM23* (upregulated in CD5^hi^; log_2_ fold change 5.50) (Table 1). Of note, in accordance with our data, *Dntt* has been shown to be downregulated in naïve Ly6C^−^ CD4^+^ T cells as compared to their Ly6C^+^ counterparts and is the most DEG between murine naïve CD5^lo^ and CD5^hi^ CD8^+^ T cells [7, 15]. Consistent with our RNAseq analysis, we observed higher *DNTT* expression in the CD5^lo^ population enriched from independent donors, whereas *ADAM23* expression was higher in the CD5^hi^ cell subset as assessed by qPCR (Fig. 4C). In addition, CD6 cell surface levels are known to correlate with CD5 expression, and CD6 is also upregulated in CD5^hi^ as compared to CD5^lo^ naïve human CD4^+^ T cells in our RNAseq dataset [28, 29]. Therefore, CD6 expression in naïve human CD5^lo^ and CD5^hi^ CD4^+^ T cells was assessed at the protein level by flow cytometry. Indeed, CD5^hi^ cells expressed higher levels of CD6 as compared to their CD5^lo^ counterparts (∼twofold difference) (Fig. 4D).

Notably, we did not detect differences in *Nur77* (*NR4A1*) expression between CD5^lo^ and CD5^hi^ naïve human CD4^+^ T‐cell subsets; this is consistent with published datasets from mice in which some, but not others report differences in *Nur77* on cells isolated from secondary lymphoid organs [7, 15, 16]. In our studies, this may be due to the heterogeneity among human samples or a muting of differences in CD4^+^ T cells in the blood, where the T cells are not receiving tonic signals from interactions with self‐peptide as they do in the secondary lymphoid organs. Indeed, differences in Nur77 protein levels among CD5^lo^ and CD5^hi^ murine naïve CD4^+^ T cells in the blood are more limited as compared to those isolated from the secondary lymphoid organs; regardless, we detect subtle, but significant, differences in NUR77 among naïve human CD4^+^ T cells from fresh, but not frozen, human blood at the protein level (Supporting Information Fig. S7). Together, these findings suggest that significant differences in gene expression and protein levels exist among naïve human CD4^+^ T cells, and that our RNAseq datasets may underestimate the extent of these differences.

Of the DEG, we identified several with known roles in the modulation of the T‐cell response such as TCR signaling regulators (*CD5, CD6, DUSP8, PLXNA4*), cosignaling molecules (*CTLA4, CD74*), and cytokines and cytokine receptors (*TGFB1*, *IL2Rb, IL4R*) (Fig. 4E). We also identified differential expression of both transcription modulators such as *NOTCH1, DAPK1, FOXN2, FOSL2, NFATC1* as well as genes involved in cell migration such as *ADAM23, ITGA1, RHOG, SLC7A5, WNT7* among naïve human CD5^lo^ and CD5^hi^ CD4^+^ T cells (Fig. 4E). Together, these data suggest that, similar to murine cells, human naïve CD5^lo^ and CD5^hi^ CD4^+^ T cells are transcriptionally distinct and that this may impact the effector potential of these cell populations.

### CD5 levels mark functionally diverse naïve CD4^+^ T‐cell subsets

**Figure 5:**
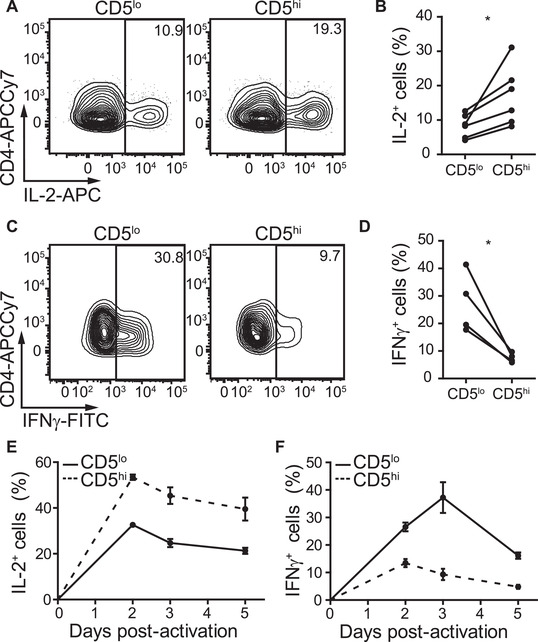
**Distinct cytokine production by CD5^lo^ and CD5^hi^ CD4^+^ human T cells**. Naïve CD45RA^+^CD27^+^CD4^+^ T cells were enriched from human PBMCs and activated under Th0 or Th1 polarizing conditions. Cytokine production was analyzed by gating on activated CD25^+^CD4^+^ T cells 2, 3, or 5 days postactivation and measured by flow cytometry. Representative flow plots (A) and proportion of IL‐2^+^ cells (B) among activated CD5^lo^ and CD5^hi^ CD4^+^ T cells in Th0 conditions at day 5 postactivation. Representative flow plots (C) and proportion of IFN‐γ^+^ cells (D) among activated CD5^lo^ and CD5^hi^ CD4^+^ T cells in Th1 conditions at day 5 postactivation. (E) IL‐2 and (F) IFN‐γ production 2, 3, and 5 days postactivation under Th1 conditions. (B and D) Dots indicate individual donors. Data are from a minimum of two independent experiments with a minimum of two donors each. *p < 0.05 as determined by paired Student's *t*‐test (two‐tailed). (E and F) Data are representative of three independent experiments with three independent donors and averaged from technical duplicates for each donor. Error bars indicate standard error of the mean.

Recent evidence suggests that differences in CD5 levels track with functional properties such as differences in interleukin‐2 (IL‐2) production and differentiation into T_reg_ [8, 10, 11]. Further, we have recently shown that a greater frequency of activated CD5^lo^ CD4^+^ T cells produce IFN‐γ as compared to their CD5^hi^ counterparts [12]. To determine if differences in CD5 levels also reveal biases in the functional contribution of human CD4^+^ T cells upon antigen recognition, we assessed cytokine production by CD5^lo^ and CD5^hi^ human CD4^+^ T cells after activation. As CD5 ligation can affect activation of human T cells [30, 31, 32], sorting cells based on CD5 levels before activation may confound the data. To address this, we first analyzed CD5 levels on sorted naïve CD5^lo^ and CD5^hi^ CD4^+^ T cells after activation. Our results demonstrate that although CD5 levels are upregulated upon activation, sorted CD5^lo^ and CD5^hi^ cells still retain differences in their CD5 levels (Supporting Information Fig. 8A and B), suggesting that stimulation of bulk naïve CD4^+^ T cells with subsequent gating on CD5^lo^ and CD5^hi^ CD4^+^ T cells postactivation is representative of daughter cells from naïve CD5^lo^ and CD5^hi^ populations. We used this information to compare the cytokine production potential of naïve human CD5^lo^ and CD5^hi^ CD4^+^ T cells in vitro. Enriched naïve CD4^+^ T cells were activated with anti‐CD3 and anti‐CD28 in the presence or absence of T helper 1 (Th1) skewing cytokines. Five days postactivation, we assessed IL‐2 or IFN‐γ production by activated CD25^+^CD4^+^ T cells with high (top 20%) or low (bottom 20%) CD5 levels (Supporting Information Fig. 8C). A greater frequency of human CD5^hi^ CD4^+^ T cells produced IL‐2 than the CD5^lo^ CD4^+^ T‐cell population in the absence of lineage skewing cytokines (Th0 condition) (Fig. 5A and B). Conversely, among Th1 polarized CD4^+^ T cells, a greater frequency of CD5^lo^ CD4^+^ T cells produced IFN‐γ as compared to their CD5^hi^ counterparts (Fig. 5C and D). It has recently been suggested that the kinetics of cytokine production among murine CD5^lo^ and CD5^hi^ CD4^+^ T cells may differ [22]. Therefore, we assessed both IL‐2 and IFN‐γ production in CD5^lo^ and CD5^hi^ CD4^+^ T cells at different time points poststimulation. We observed similar biases in cytokine production among CD5^lo^ and CD5^hi^ CD4^+^ T cells at days two and three poststimulation as at day five (Fig. 5E and F). However, we cannot rule out that this pattern may change at earlier or later time points. Overall, our data suggest that functional heterogeneity, in terms of effector cytokine production biases, exists in naïve CD4^+^ T cells prior to activation and can be parsed based on CD5 levels.

## Discussion

Naïve CD4^+^ T cells have long been considered homogenous in terms of their functional potential after antigen encounter. However, several recent studies using murine models have demonstrated that functional biases exist prior to antigen challenge and can be parsed based on markers that reflect the strength of TCR engagements with self‐pMHC on individual murine CD4^+^ and CD8^+^ T cells [7, 8, 9, 10, 11, 12]. Our current study identifies similarities in CD5 regulation in mouse and human naïve CD4^+^ T‐cell populations and demonstrates that cell surface levels of CD5 can be used to identify T cells with distinct transcriptional profiles and functional biases. Further, owing in part to the absence of available tools for human studies, much of our understanding of T‐cell development and function stems from findings in murine models. Given our report that many aspects of CD5 regulation are conserved between mouse and human naïve CD4^+^ T cells, our study lends credence to other premises extrapolated from murine models including T‐cell development and function in humans.

One important difference between the murine and human samples used for our functional studies is the source of the T cells. While splenic naïve CD4^+^ T cells were isolated for functional analyses of cells of murine origin, analysis of their human counterparts was largely performed on naïve CD4^+^ T cells isolated from PBMCs. Cells in the blood are dissociated from the tonic signaling obtained in secondary lymphoid organs, and the duration of time naïve T cells spend in the blood may differ between mice and humans [33, 34]. As such, some differences in gene expression or functional bias observed in murine samples may be more subtle in human cells isolated from the blood. Furthermore, the distribution of CD5 levels, as assessed by analysis of CV, is significantly narrower on human CD4^+^ T cells as compared to the corresponding murine cell populations which may limit the extent of functional biases observed in human cells. Despite these differences in T‐cell origin between murine and human T cells and the more limited breadth of CD5 expression on human naïve CD4^+^ T cells, we still observe considerable differences in the functional potential of naïve human CD5^lo^ and CD5^hi^ CD4^+^ T cells.

Differences in the subthreshold self‐reactivity of T cells that reflect biases in effector potential are relevant to human health. In addition to the conventional CD4^+^ T‐cell population that we describe here, there is also evidence that CD5 levels may identify human T_reg_ subsets with distinct functions. More specifically, CD5^hi^ human T_reg_ preferentially express ICOS, FOXP3, GITR, and CTLA4 similar to a subpopulation of these cells in mice that are more protective in the context of tissue‐specific autoimmunity and immune homeostasis as compared to their CD5^lo^ counterparts [35, 36]. Notably, we have previously shown that both conventional and T_reg_ cells in neonates express higher levels of CD5 on their cell surface as compared to adult T cells due to differences in thymic selection thresholds that generate a T‐cell repertoire with higher basal self‐reactivity [37]. Given that the strength of these tonic TCR interactions with self‐pMHC influence T‐cell function, it is possible that differences in CD5 levels on neonatal versus adult T cells explain, in part, some of the differences in their T‐cell response that render neonates more susceptible to infection.

Establishing CD5 as a reliable marker of functional heterogeneity in human naïve CD4^+^ T cells sets a foundation for developing tools to isolate T cells with specific characteristics to improve adoptive T‐cell therapy protocols. Typically, total T‐cell pools are used for ex vivo expansion and transfer into patients. However, many patients relapse, and this often correlates with an inability to detect functional transferred T cells. In this context, differences in CD5 levels could be used to identify T cells with attractive functional biases. For example, enhanced IFN‐γ production by CD5^lo^ T cells could induce epitope spreading and lead to more efficient tumor rejection [38]. In mouse studies of naïve CD4^+^ T cells, CD5^hi^ T cells preferentially expand in vitro and in vivo in response to acute antigen challenge, but they are also more prone to T_reg_ differentiation [8, 9]. Of the phenotypes we tested in human T cells, all were consistent with those observed in mice. Thus, it is conceivable that although CD5^hi^ T cells may predominate when expanded in culture, it is in fact T‐cell populations with low levels of CD5 that confer an advantage for functionality and persistence. Whether directly modifying CD5 levels on human T cells will also be relevant for therapeutic purposes is less obvious, as it is not clear to what extent differences in the levels of CD5 on the surface of naïve T cells directly influence their function after activation. A recent whole‐genome CRISPR screen identified CD5 as an important modulator of human T‐cell proliferation after activation in vitro and positions CD5 as a potential checkpoint to enhance T‐cell expansion for adoptive cell therapies [39]. While completely removing the CD5 “brake” on T‐cell activation will undoubtedly improve T‐cell expansion, whether these cells would remain functionally competent in the longer term is not known [40]; perhaps a more subtle parsing of optimal T‐cell populations based on cell surface CD5 levels will provide better therapeutic results.

## Material and methods

### Study approval

Human thymus was obtained after written informed parental consent and in accordance with Research Ethics Committee guidelines at Centre Hospitalier Universitaire Sainte‐Justine and Maisonneuve–Rosemont Hospital. Human peripheral blood was obtained from donors after written informed consent and in accordance with Research Ethics Committee guidelines at the Maisonneuve–Rosemont Hospital or University Medical Center Utrecht. All Research Ethics Committee guidelines were in accordance with the Declaration of Helsinki principles. All animal protocols were approved by the local Animal Care Committee in accordance with the Canadian Council on Animal Care.

### Human tissue procurement and preparation

Human thymus samples were procured from pediatric patients undergoing corrective cardiac surgery. A small piece (0.5 cm^2^) of tissue was removed, and a single cell suspension of thymocytes was prepared in 10 mL of PBS + 2% fetal bovine serum (GE Life Sciences) using a glass tissue homogenizer followed by RBC lysis with Ack lysis buffer (0.15 M NH_4_Cl, 10 mM KHCO_3_, 0.1 mM Na_2_EDTA) before use. Human peripheral blood was obtained from healthy donors. Mononuclear cells were isolated by density gradient centrifugation over Ficoll–Paque (GE Healthcare) and used fresh or cryopreserved until use. DRB1* HLA alleles were typed by Sanger sequencing at the Molecular Diagnostic Laboratory of the Hospital Maisonneuve–Rosemont. Cryopreserved PBMCs were thawed and incubated for 1‐2 h at 37°C before use.

### Mouse tissue

C57BL/6 mice were purchased from The Jackson Laboratory and maintained in a specific pathogen‐free environment at the Maisonneuve–Rosemont Hospital Research Center. Thymic and peripheral lymphoid tissues were harvested from 6‐ to 12‐week‐old mice. Both male and female mice were used. Single‐cell suspensions were prepared manually with glass tissue homogenizers followed by RBC lysis as above before use.

### Antibodies and flow cytometry

Anti‐human TCRαβ (IP26), CD3 (UCTH1), CD4 (OKT4, RPA‐T4), CD8α (RPA‐T8), CD5 (UCHT2), CD45RA (HI100), CD27 (M‐T271), CD69 (FN50), CD62L (DREG‐56), CD25 (BC96), CD127 (HCD127), IFN‐γ (4S.B3) and anti‐mouse CD4 (GK1.5), CD8α (53‐ 6.7), TCRβ (H57‐597), CD5 (53‐7.3), CD69 (H1.2F3), CD25 (PC61), CD44 (IM7) and CD62L (MEL‐14) antibodies were purchased from BioLegend. Anti‐human IL‐2 (MQ1‐17H12), CD5 (L17F12), CD27 (O323), and Foxp3 (150D/E4) antibodies, and anti‐mouse Nur77 (12.14) were purchased from ThermoFisher. Anti‐human CD3 (SP34‐2) and CD31 (WM59) were purchased from BD Biosciences. To block nonspecific antibody binding, murine and human cells were incubated with 2.4G2 supernatant or Human TruStain FcX (BioLegend), respectively, at 4°C for 10 min except for experiments comparing CD5 MFI on naïve versus memory human CD4^+^ T cells. Cells were then stained with either Live/Dead Zombie (BioLegend) or Live/Dead Fixable Violet Stain (Life Technologies) according to manufacturer's instructions followed by incubation with cell surface antibodies for 20 min at 4°C. For intracellular staining, cells were fixed and permeabilized using the Cytoperm/Cytofix kit (BD Biosciences), and for intranuclear staining, the Foxp3 Staining Kit (eBioscience) was used according to the manufacturers’ protocols. PE‐Quantibrite beads (BD Biosciences) were used to normalize expression for data collected on different days. Cells were analyzed on a Fortessa X‐20 flow cytometer (BD Biosciences). Cell sorting was performed using the FACS Aria III instrument (BD Biosciences). FlowJo software (BD Biosciences) was used for flow cytometry data analysis. Flow cytometry and cell sorting were performed in line with published guidelines [41].

### Ex vivo tetramer analysis

HLA‐DRB1*01:01 restricted B. *Anthracis* Protective Antigen peptide 713–732 (KLPLYISNPNYKVNVYAVT) and HLA‐DRB1*04:01 restricted HIV‐1 Gag p24 peptide 164–183 (AFSPEVIPMFSALSEGATPQ) were custom synthesized to greater than 95% purity (GenScript). Peptide loaded biotinylated monomers were obtained from the National Institutes of Health Tetramer Core Facility. The peptide‐loaded monomers were subsequently cross‐linked with PE‐labeled streptavidin (Invitrogen) or BV421‐labeled streptavidin (BioLegend) for at least 4 h at 4°C to produce peptide tetramers. PBMCs (150‐250 × 10^6^) were thawed and rested at 37°C for 2 h before staining with Live/Dead Zombie viability dye. Tetramer staining was carried out for 1 h (HIV‐1) or 2 h (*B. anthracis*) at room temperature using 10 μg/mL of tetramer. Cell surface antibodies were added in the last 20 min of the tetramer stain. Tetramer tagged cells were magnetically labelled by adding anti‐PE beads (Miltenyi Biotec) and enriched by passing through a magnetic column (Miltenyi Biotec). Cells were acquired on a Fortessa X‐20 flow cytometer (BD Biosciences) and analyzed using FlowJo software (BD Biosciences).

### T‐cell enrichment

Naïve CD4^+^ T cells were enriched from PBMCs by negative selection using the EasySep Naïve CD4 T‐Cell Isolation Kit (StemCell Technologies). The purity of the naïve CD4^+^ T‐cell fraction, assessed by analysis of live CD45RA^+^CD27^+^ CD4^+^ T cells, was >90% for all the samples. Total T cells were enriched from PBMCs by negative selection using the EasySep Human T‐cell Enrichment Kit (StemCell Technologies).

### T‐cell activation

Cells were cultured in nonadherent flat bottom 96‐well plates in complete Roswell Park Memorial Institute media (Wisent) supplemented with 10% fetal bovine serum (GE Life Sciences), 2 mM l‐glutamine (Wisent), 100 IU penicillin (Wisent), 100 μg/mL streptomycin (Wisent), 50 mM 2‐β‐mercaptoethanol (Bio‐Rad Laboratories). A total of 0.5‐1 × 10^6^ cells per well were stimulated with different concentrations of plate‐bound anti‐CD3 (Human: OKT3, eBioscience; Mouse: 145‐2C11; BioLegend). For analysis of cytokine production, enriched naïve CD4^+^ T cells were stimulated with plate‐bound anti‐CD3 (5 μg/mL, OKT3, eBioscience) and soluble anti‐CD28 (2 μg/mL, CD28.2, BioLegend). For Th1 polarization, media was supplemented with IL‐12 (2.5 ng/mL, Peprotech), anti‐IL‐4 (5 μg/mL, MP4‐25D2, BioLegend), and anti‐IL‐10 (5 μg/mL, JES3‐9D7, BioLegend). At 3 days postactivation, half of the media was removed and fresh media with IL‐2 (40 ng/mL, Peprotech) for Th0 and IL‐2 (40 ng/mL, Peprotech), anti‐IL‐4 (4 μg/mL, BioLegend), and anti‐IL‐10 (4 μg/mL, BioLegend) for Th1‐skewing conditions was added to the cultures. Cells were harvested 5 days postactivation and cytokine production analyzed. For intracellular cytokine staining, in vitro activated cells were restimulated with PMA (50 ng/mL, Sigma Aldrich) and ionomycin (1 μg/mL, Calbiochem) in the presence of Golgi Plug (BD Biosciences) for 4 h at 37°C. Staining was performed as described above.

### RNAseq

Cryopreserved PBMCs were thawed and incubated for 1 h at 37°C followed by T‐cell enrichment. Actinomycin D (5 μg/mL, Sigma‐Aldrich) was added to cell suspension media during T‐cell enrichment and staining to inhibit transcription [42]. Naïve CD27^+^CD45RA^+^CD25^−^CD4^+^ T cells were sorted directly into Trizol LS (Thermo Fisher Scientific) based on CD5 expression (top and bottom 15%). RNA sequencing was performed at the Genomics Platform of the Institute for Research in Immunology and Cancer. Library preparation was performed with the Illumina Nextera XT kit before single‐end RNAseq using the Illumina NextSeq500 platform for 75 cycles (NextSeq500 Mid Output Kit). Sequences were trimmed for sequencing adapters and low quality 3' bases using Trimmomatic version 0.35 and aligned to the reference human genome version GRCh38 (gene annotation from Gencode version 24, based on Ensembl 88) using STAR version 2.5.1. FPKM values for gene and transcript level expression for these stranded RNA libraries were obtained both as readcounts directly from STAR as well as computed using RSEM. DESeq2 version 1.18.1 was then used to normalize gene readcounts. Benjamini–Hochberg *p*adj were used to compute differential expression between groups with DEG defined as those having a *p*adj value of ≤0.05 (for heat maps, a *p*adj value of ≤0.1 was used). Heat maps were generated using the *pheatmap* package (version 1.0.12) within the open source statistical program R Project (https://www.R‐project.org/; https://CRAN.R‐project.org/package = pheatmap). The raw data for RNAseq is available at the NIH Gene Expression Omnibus site (accession # GSE148662).

### Quantitative RT‐PCR

Total RNA was isolated from sorted naïve CD5^hi^ and CD5^lo^ (top and bottom 20%) CD4^+^ T cells with Trizol (Invitrogen) and DNase‐I (Invitrogen) treated to remove genomic DNA. cDNA was prepared using M‐MLV reverse transcriptase (Invitrogen) as per manufacturer's instructions. For qPCR analysis, cDNA was amplified using an ABI PRISM 7700 System and TaqMan reagents for *ADAM23* (Applied Biosystems) or SYBR Green (Thermo Fisher) for *DNTT*. The following primers were used: *DNTT* (Forward: CCGTCAGTGTGCTGGTTAAAG; Reverse: AGTTGCTCTTCATCCTCTGTTGA; amplicon 150bp), *ADAM23* (Hs00187022_m1; amplicon 110 bp), and *HPRT* (Forward: CCTGGCGTCGTGATTAGTGAT; Reverse: AGACGTTCAGTCCTGTCCATAA; amplicon 132 bp and Hs03929098_m1; amplicon 159 bp). The assays were run in triplicate using water as a negative control, and the average *Ct* values for the housekeeping gene *HPRT* and target genes in CD5^lo^ and CD5^hi^ cells were determined. Fold change of gene expression was calculated as log_2_‐transformed 2^delta‐delta *Ct* values.

### Statistics

Statistical analyses were performed using GraphPad Prism (GraphPad Software). Paired Student's *t*‐tests were performed unless otherwise indicated. Statistical significance is indicated by *p* values: *p < 0.05, **p < 0.01, ***p < 0.001, ****p < 0.0001. ns indicates nonsignificant values. Coefficient of Variation analysis (robust CV) was performed in FlowJo.

## Author contributions

AS, MEL, MD, JNM, NV, and HJM designed experiments. AS, MEL, MD, MF, and NV executed experiments. EH, SJV, and JSD provided reagents and human tissue samples as well as critical input. AS, MEL, MF, MD, and JNM generated figures. AS and HM wrote the manuscript, and all authors revised the manuscript.

## Conflict of interest

The authors have declared that no commercial or financial conflict of interest exists.

### Peer review

The peer review history for this article is available at https://publons.com/publon/10.1002/eji.202048788

**Abbreviations**
CV: coefficient of variation
DEG: differentially expressed genes
DN: double negative
DP: double positive
IFN‐γ: interferon‐gamma
IL‐2: interleukin‐2
pMHC: peptide presented by major histocompatibility complex molecules
RFI: relative fluorescence intensity
SP: single positive
Th1: T helper 1

## Supporting information

Supporting Information

## Data Availability

The RNAseq data have been deposited in the NIH/NCBI Gene Expression Omnibus and are accessible through GEO Series accession number GSE148662. All other data that support the findings of this study are available from the corresponding author upon request.
