# Supplementary material for: CD5 levels define functionally heterogeneous populations of naïve human CD4+ T cells
Source: Eur J Immunol. 2021 Mar 19;51(6):1365–76. doi: 10.1002/eji.202048788 (PMC8251777; doi:10.1002/eji.202048788)
Supplement: Supplementary file 1 — Supporting Information [file EJI-51-1365-s001.pdf]

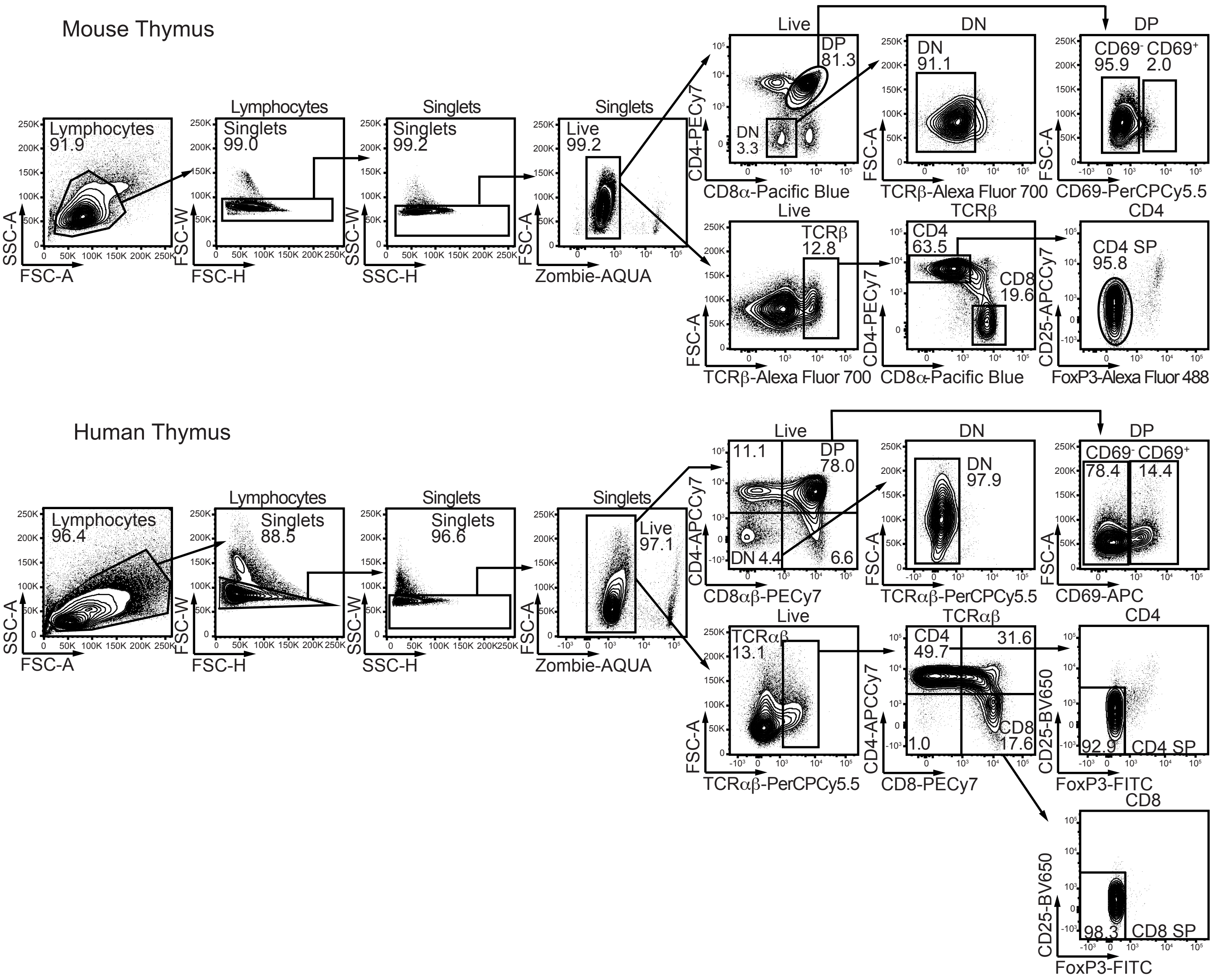

Supporting Information Figure 1. Gating strategy for mouse and human thymocyte subsets. Cells were first gated on a lymphocyte gate, then singlets and live cells. T cell developmental intermediates were gated as: live $CD4^{-}CD8^{-}$ $TCR\beta^{-}$ (mouse) or $TCR\alpha\beta^{-}$ (human) double negative (DN), live $CD4^{+}CD8^{+}CD69^{-}$ pre-selection, live $CD4^{+}CD8^{+}CD69^{+}$ post-selection double positive (DP), and $TCR\beta^{high}$ (mouse) or $TCR\alpha\beta^{high}$ (human) $Foxp3^{-}CD25^{-}$ $CD4^{+}$ and $CD8^{+}$ single positive (SP) thymocytes. Numbers adjacent to outlined areas represent frequency of cells.

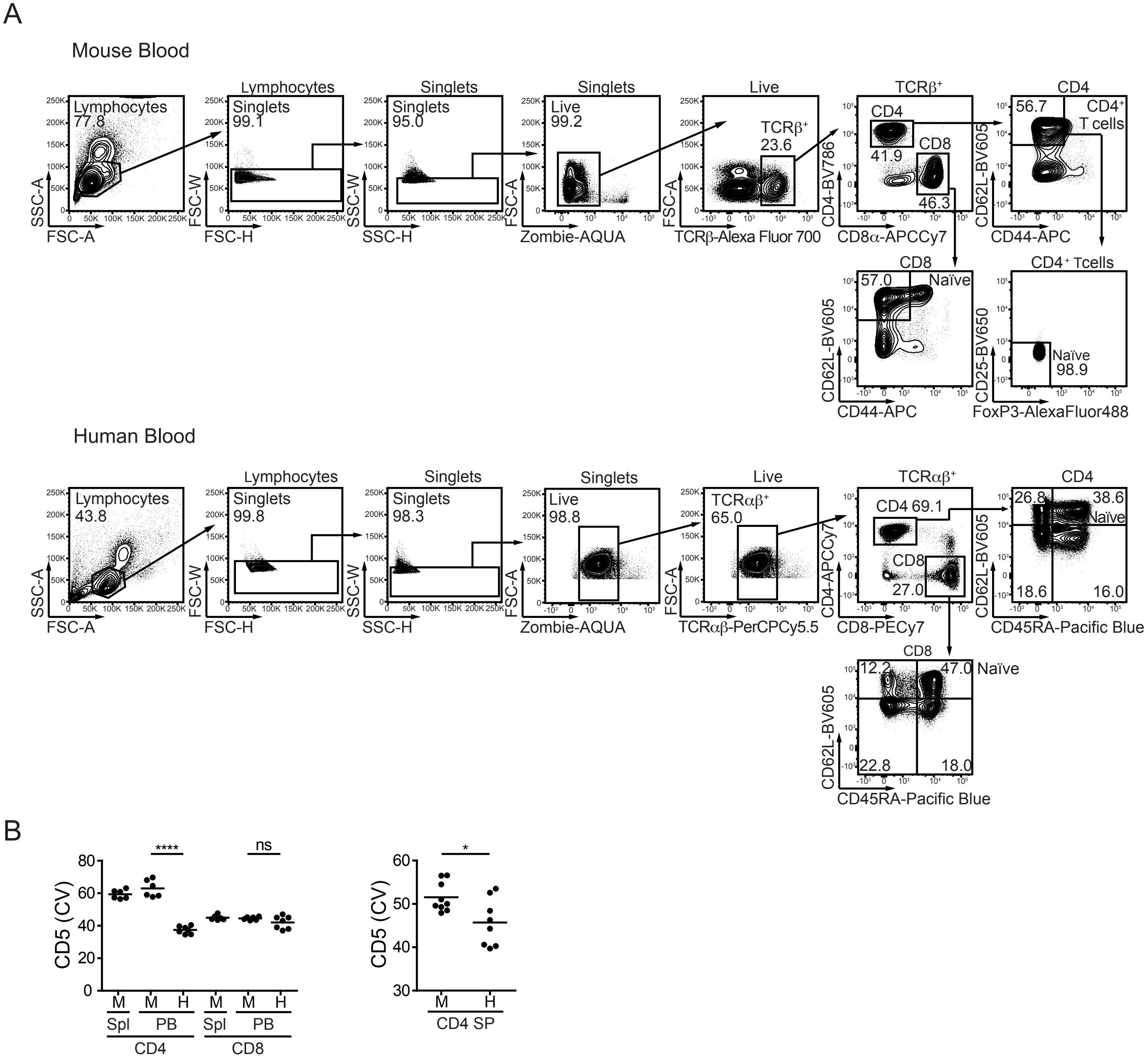

Supporting Information figure 2. The distribution of CD5 expression on naïve CD4$^+$ T cells is narrower in humans than mice. (A) Gating strategy for naïve CD4$^+$ and CD8$^+$ T cells from mouse and human blood samples. Cells were first gated on lymphocytes, then singlets and live cells. Naïve cells were gated as TCRβ$^+$ CD44$^-$CD62L$^+$CD25$^-$Foxp3$^-$CD4$^+$ T cells and TCRβ$^+$CD44$^-$CD62L$^+$CD8$^+$ T cells for mouse blood or TCRαβ$^+$ CD45RA$^+$CD62L$^+$ CD4$^+$ and CD8$^+$ T cells for human PBMCs. Numbers adjacent to outlined areas represent frequency of cells. (B) Left panel: CV of CD5 fluorescence on naïve CD4$^+$ and CD8$^+$ T cells from spleen (Spl) and peripheral blood (PB) of mouse (CD44$^-$CD62L$^+$) and human (CD45RA$^+$CD62L$^+$) samples. Dots represent individual mouse (M) or human (H) samples. Data are from a minimum of two independent experiments. Right panel: CV of CD5 fluorescence on TCRβ^{high} (mouse) or TCRαβ^{high} (human) CD4$^+$ SP cells from mouse (M) and human (H) thymus. Dots represent individual mouse or human thymus. Data are from a minimum of three independent experiments. *P<0.05, ****P<0.0001, ns, non-significant as determined by unpaired Student's t test (two-tailed).

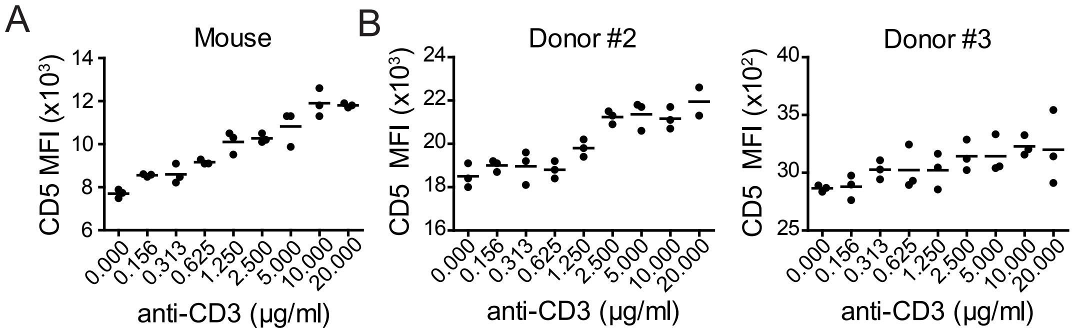

Supporting Information figure 3. TCR dose dependent upregulation of CD5 in mouse and human CD4$^+$ thymocytes. MFI of CD5 gated on live CD69$^+$CD4$^+$ thymocytes activated with the indicated concentrations of anti-CD3 for 18 h is shown for mouse (A) and two additional human donors (B). Dots represent technical triplicates from an individual mouse or human thymus. Data is representative of one experiment from three independent experiments.

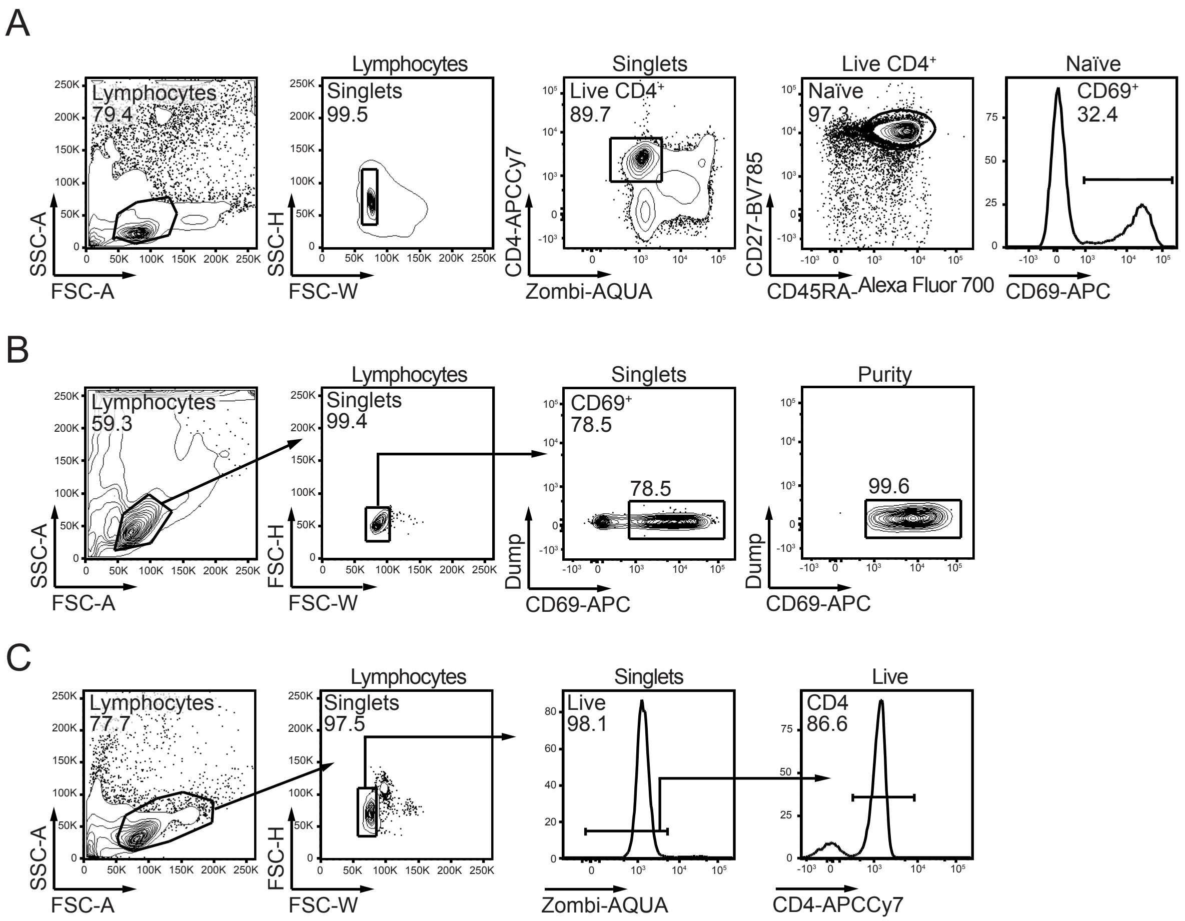

Supporting Information figure 4. Activation and sorting of human CD4⁺ T cells. (A) Gating strategy for CD69 and CD5 levels on enriched naïve human CD4⁺ T cells after activation. Cells were first gated on lymphocytes, then singlets and live cells. Naïve cells were gated as CD45RA⁺CD27⁺ CD4⁺ T cells for CD69 analysis, or CD45RA⁺CD27⁺CD69⁺ CD4⁺ T cells for CD5 analysis. (B) Sorting strategy for CD69⁺ T cells 24 h after activation of enriched human naive CD4⁺ T cells. Cells were first gated on live lymphocytes prior to CD69⁺ cells. The purity of the sorted population was routinely >98%. (C) Gating strategy for CD69 and CD5 analysis on T cells on pre-sort non-stimulated cells as well as after 24 h and post-sort cells for up to 9 d. Numbers adjacent to outlined areas represent frequency of cells.

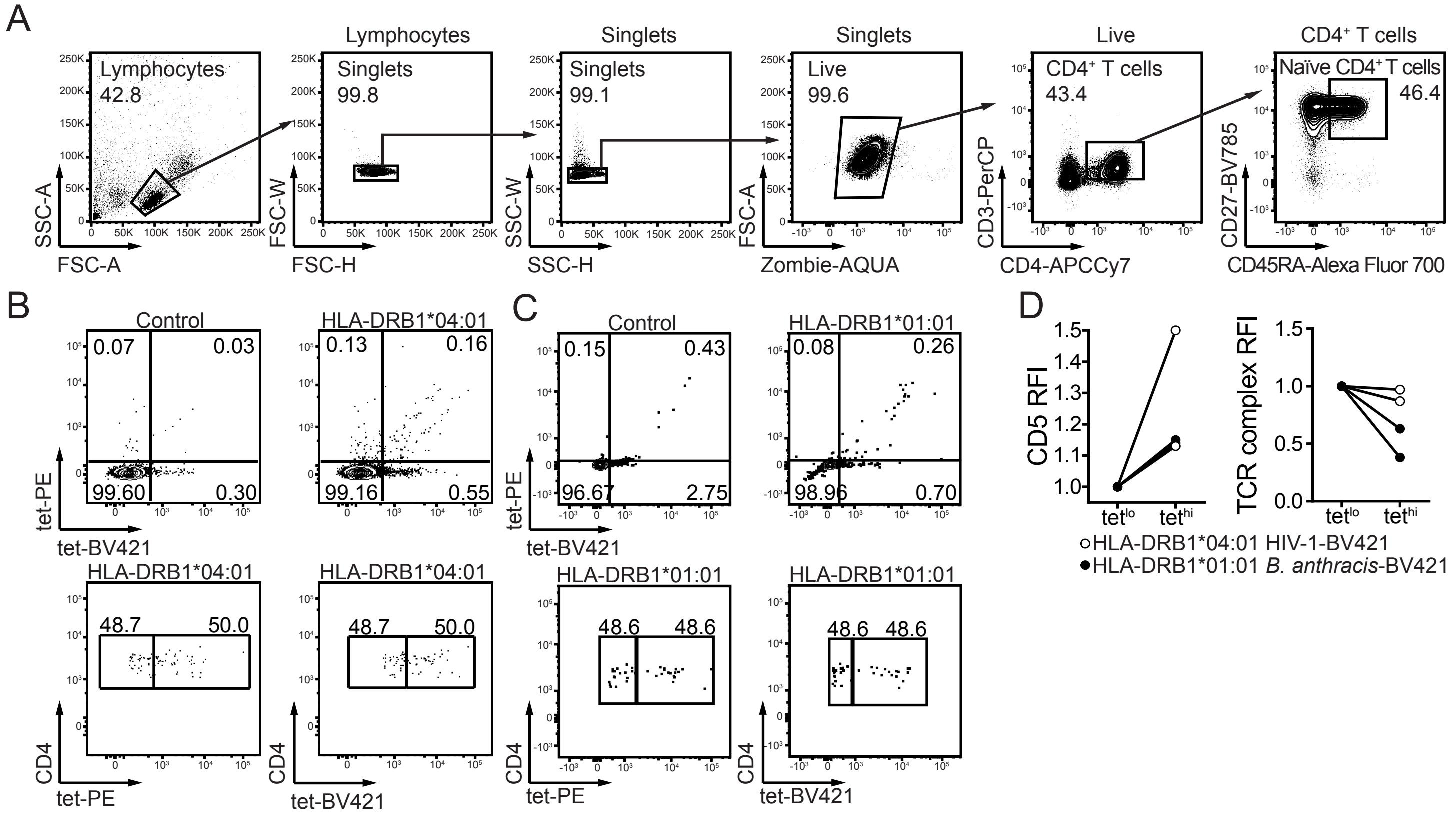

Supporting Information figure 5. CD5 levels on human foreign-pMHC specific CD4⁺ T cells. Human PBMCs were stained with BV421- and PE-labeled tetramers followed by cell surface antibodies and PE enrichment of tetramer⁺ cells. BV421⁺PE⁺ double-positive naïve CD45RA⁺CD27⁺CD4⁺ T cells were gated on the top (tet^{hi}) and bottom (tet^{lo}) 50% of BV421-tetramer staining intensity, and cell surface levels of CD5 and TCR or CD3 expression analyzed. (A) Representative gating strategy for naïve CD4⁺ T cells for tetramer analysis. Cells were first gated on lymphocytes, then singlets and live cells. CD4⁺ T cells were subsequently gated on live cells as CD3⁺CD4⁺ followed by gating on CD45RA and CD27 to identify naïve CD45RA⁺CD27⁺CD4⁺ T cells prior to analysis of tetramer staining. Representative flow plots depicting HIV-1 (B) or *B. anthracis* (C) peptide-loaded tetramer labeling and gating strategy for tet^{lo} and tet^{hi} subsets gated either on PE- or BV421-tetramer staining intensity. PBMCs from a non-HLA*04:01 or non-HLA*01:01 donor were used as controls for HIV-1 and *B. anthracis* tetramer labeling, respectively. (D) Relative levels of CD5 (left panel) or the TCR complex (right panel) on tet^{lo} and tet^{hi} cells gated on 50% of BV421-tetramer staining intensity is shown for HIV-1 and *B. anthracis* peptide loaded tetramers for two HLA-DRB1*04:01 and HLA-DRB1*01:01 donors, respectively. Data are from four independent experiments with two experiments for each tetramer. Dots represent individual donors. RFI was calculated by normalizing to the MFI of CD5 or TCR complex molecules on tet^{lo} cells for each condition.

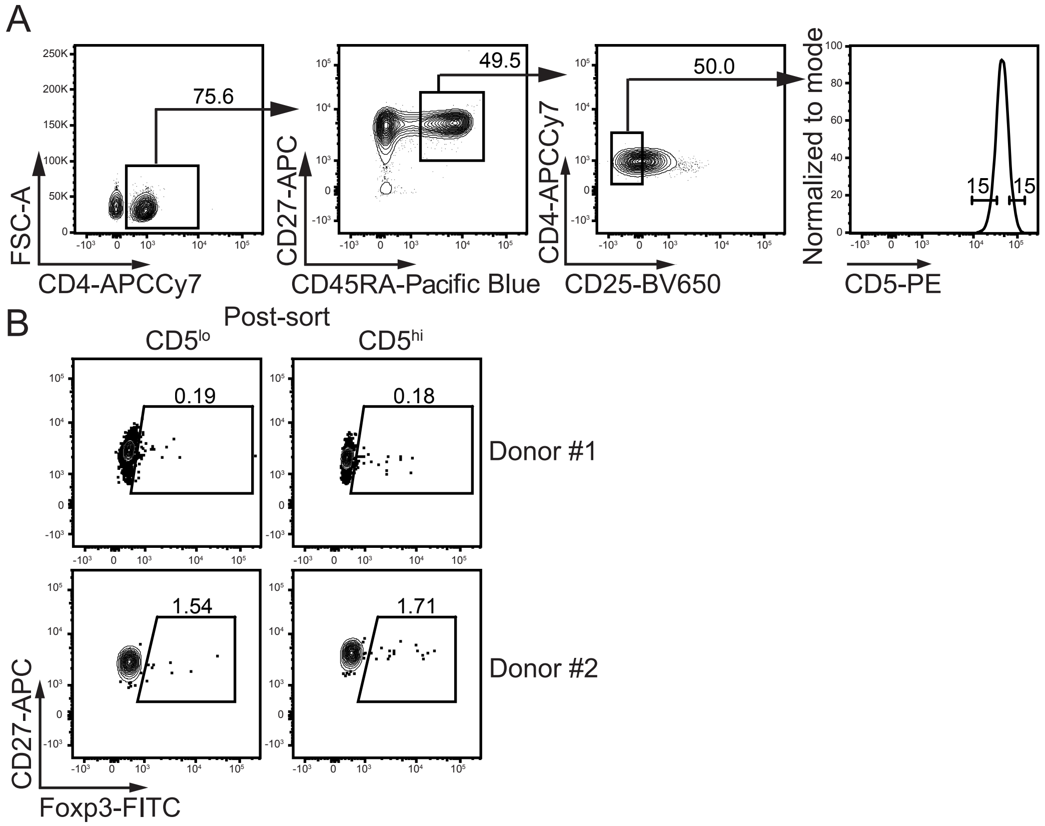

Supporting Information figure 6. Gating strategy and purity of sorted CD5^{lo} and CD5^{hi} naïve human CD4^{+} T cells for transcriptomic analysis. (A) Gating strategy for sorting CD5^{lo} and CD5^{hi} naïve CD45RA^{+}CD27^{+}CD25^{-} CD4^{+} T cells. Total T cells enriched from PBMCs were used for sorting. (B) Flow plots depicting proportion of Foxp3^{+} T cells among sorted CD5^{lo} and CD5^{hi} naïve CD4^{+} T cells. Numbers adjacent to outlined areas represent frequency of cells.

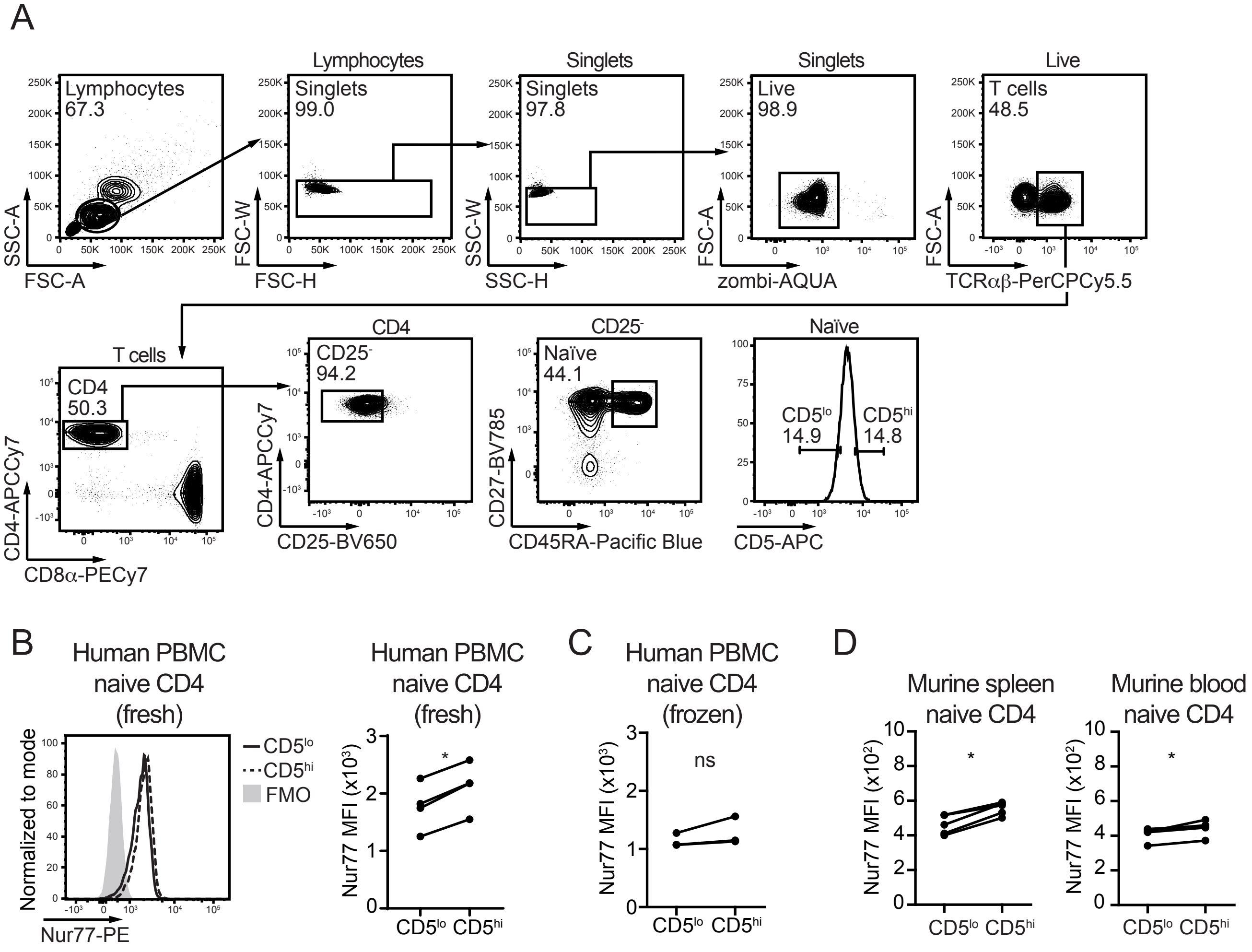

Supporting Information figure 7. Differences in Nur77 levels on human CD5^{lo} and CD5^{hi} CD4^{+} T cells. (A) Gating strategy for analysis of Nur77 levels on CD5^{lo} and CD5^{hi} naïve human CD4^{+} T cells from PBMC. Cells were first gated on lymphocytes, then singlets and live cells. Naïve cells were gated as TCRαβ^{+}CD45RA^{+}CD27^{+} CD4^{+} T cells prior to the top and bottom 15% of CD5, for Nur77 analysis. Numbers adjacent to outlined areas represent frequency of cells. (B) Representative histogram of Nur77 on CD5^{lo} and CD5^{hi} naive CD4^{+} T cells (left panel) and mean fluorescence intensity (MFI) of Nur77 (right panel) from fresh human PBMC samples. FMO, fluorescence minus one. (C) MFI of Nur77 on naive CD4^{+} T cells from cryopreserved human PBMC samples. (D) MFI of Nur77 on CD5^{lo} and CD5^{hi} (bottom and top 15%) naive (CD62L^{+}CD44^{-}) murine CD4^{+} T cells harvested from the spleen (left panel) or blood (right panel). Data represent a minimum of three donors. Dots represent individual donors or mice; lines connect paired data for each sample. *$P<0.05$ as determined by a paired Student's t test (two-tailed). ns, not significant.

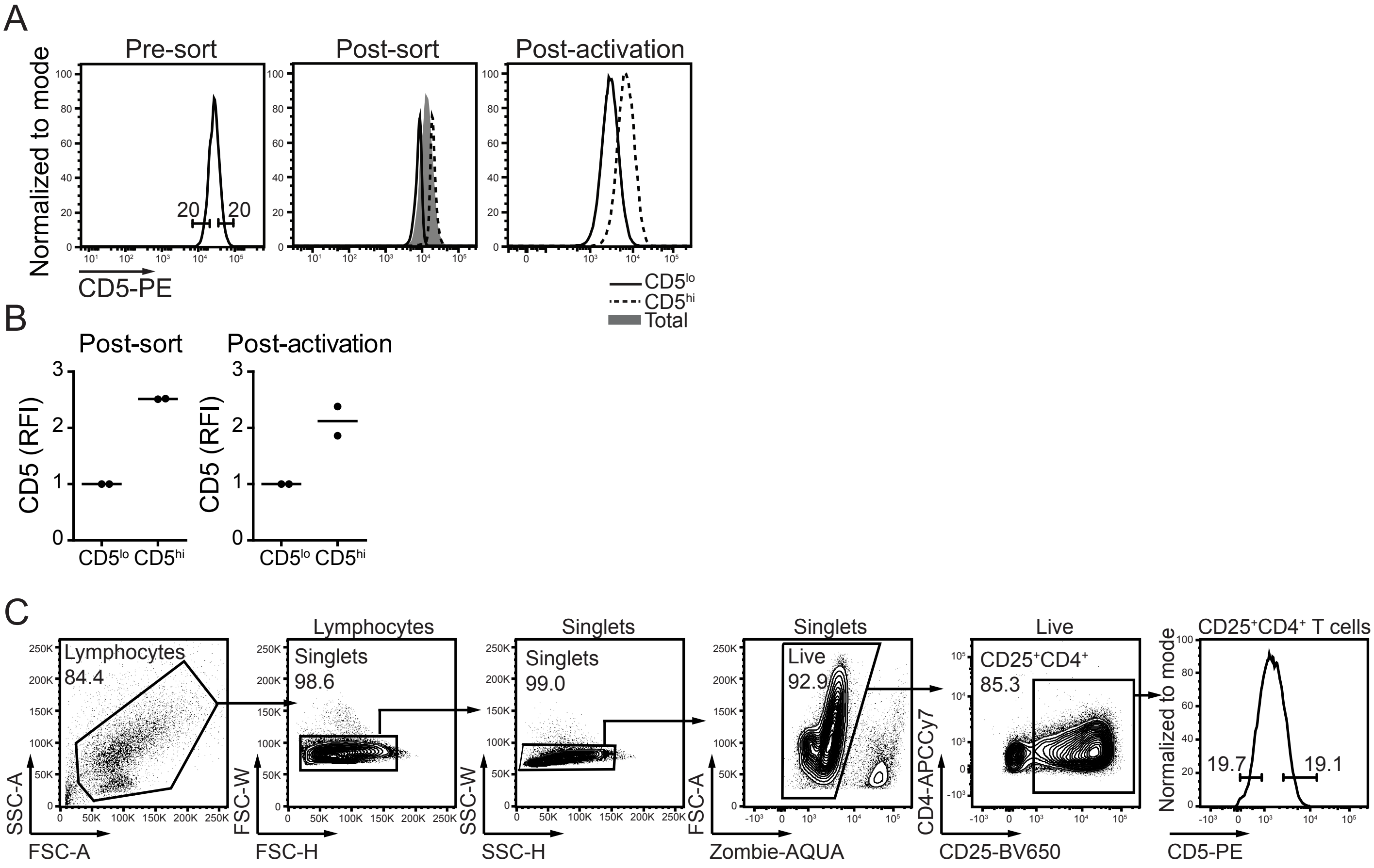

Supporting Information figure 8. Sorted naïve CD5^{lo} and CD5^{hi} cells retain differences in CD5 levels post-activation. Naïve CD45RA^{+}CD62L^{+}CD4^{+} T cells were sorted based on CD5 expression (top and bottom 20%) and activated under Th0 conditions. 5 d post-activation, cells were harvested and CD5 levels analyzed. (A) Histograms depicting CD5 levels on pre- and post-sort naïve CD4^{+} T cells and activated CD25^{+}CD4^{+} T cells 5 d post-activation. (B) Post-sort (left panel) and post-activation (right panel) CD5 RFI calculated by normalizing to the CD5 MFI of CD5^{lo} cells for each condition. Dots represent individual donors. Data is from two independent experiments with one donor each. (C) Representative strategy for gating activated (CD25^{+}) CD5^{lo} and CD5^{hi} CD4^{+} T cells to assess cytokine production. Cells were first gated on a lymphocyte gate, then singlets and live cells. Activated cells were then identified as CD25^{+} and gated on top and bottom 20% of CD5 expression.
